# FEASIBILITY OF THE NUDGELTC CLINICAL DECISION SUPPORT APPLICATION

**DOI:** 10.1093/geroni/igad104.2290

**Published:** 2023-12-21

**Authors:** Alyce Ashcraft, Donna Owen, John Culberson

**Affiliations:** Texas Tech University Health Sciences Center, Lubbock, Texas, United States; TTUHSC, Lubbock, Texas, United States; TTUHSC, Lubbock, Texas, United States

## Abstract

Long-term-care residents are at risk for under reporting of urinary tract infections due to insufficient point-of-care testing and indirect communication. Aim: Examine the feasibility of a Clinical Decision Support application (NudgeLTC) designed to guide Licensed Vocational Nurses (LVNs) in data collection to improve communication confidence and satisfaction between LVN and Primary Care Provider (PCP). Sample size: 4 LVNs and 4 PCPs across 10 events. Qualitative description of interview comments and NudgeLTC output documented data collection and communication between LVN and PCP within the app. Forced choice questions about resident signs, symptoms, and laboratory values guided LVN data collection. Information was collected and stored within NudgeLTC. A summary was relayed directly to the PCP and followed with a PCP response to the LVN. Questionnaires included visual analogue scales for confidence (LVNs M=81.6; PCPs M=100 [0 low, 100 high]), satisfaction (LVNs M=7.8; PCPs M=10 [0 low, 10 high]), and the Systems Usability Scale (LVNs M=85.4; PCPs M=100 [0 poor, 100 high]). The NudgeLTC summary included resident assessment, vital signs, and lab values. Particular signs and symptoms noted included indwelling catheter and confusion. Select Challenges: PCP received LVN message and data, but was unable to respond via app; LVN stated the app did not work as “expected.” Transmission issues occurred and were resolved. Asynchronous communication was valued and facilitated confidence in resident data. Difficulties with data collection may, in part, be due to discomfort with technology or infrequent use of electronic charting. Future studies should focus on improving point-of- care data collection.

